# Endoscopic Third Ventriculostomy with Choroid Plexus Cauterization in Infantile Hydrocephalus: An Experience from Mali

**DOI:** 10.1159/000529453

**Published:** 2023-02-08

**Authors:** Oumar Diallo, Mahamadou Dama, Landry Konan, Oumar Coulibaly, Daouda Sissoko, Abdoulaye Hima Maiga

**Affiliations:** ^a^Department of Neurosurgery, Hospital of Mali, University of Sciences, Technics and Technology of Bamako (USTTB), Bamako, Mali; ^b^Department of Neurosurgery CHU Yopougon, University Felix Houphouët Boigny, Abidjan, Ivory Coast; ^c^Department of Neurosurgery, General Hospital of Niamey, University Abdou Moumouni, Niamey, Niger

**Keywords:** Endoscopic third ventriculostomy, Choroid plexus cauterization, Hydrocephalus, Pediatric hydrocephalus, Outcomes

## Abstract

**Introduction:**

Pediatric hydrocephalus is a common disease in sub-Saharan Africa. In Mali, 350–400 new cases are diagnosed in our center yearly. With a total land mass of 1,241,000 km<sup>2</sup>, patients in remote areas must travel up to 1,500 km to access neurosurgical care. Hence, treatment and follow-ups of “shunted” patients are difficult. In this context, endoscopic third ventriculostomy with choroid plexus cauterization (ETV/CPC) provides an opportunity for an affordable and less constraining treatment for hydrocephalus children under 12 months of age.

**Methods:**

We performed a retrospective analysis of ETV/CPC performed on infants from July 2013 to January 2015. Patients were followed postoperatively on day 15, month 6, and month 12. Statistical analysis was conducted using Prism 9 GraphPad software. ETV successes were categorized according to the patient's age into 3 groups: ≤3 months, 3–6 months, and 6–12 months. Statistical significance was defined at *p* < 0.05.

**Results:**

During the study period, 199 patients were included with 40% of patients aged between 0 and 6 months. The head circumference ranged from 35 cm to 79 cm. The etiology was congenital malformation in 55%. ETV/CPC was a success in 69% of 6- to 12-month-old patients, 54% in the 3- to 6-month-old patients, and 29% in ≤3-month-old patients. Overall, 94 (47%) patients were successfully treated without a shunt. The postoperative infection rate was 1% and mortality at 12 months was 8%.

**Conclusion:**

In a low-income environment such as Mali, ETV/CPC stands as a viable and alternative treatment option for pediatric hydrocephalus patients; our findings suggest that age is an important factor in predicting ETV success.

## Introduction

Pediatric hydrocephalus is a common condition with a worldwide annual incidence of nearly 400,000 patients. In a recent study, the pooled incidence of congenital hydrocephalus was highest in Africa and Latin America (145 and 316 per 100,000 births, respectively) and lowest in the USA/Canada (68 per 100,000 births) (*p* for interaction <0.1). The incidence was higher in low- and middle-income countries (123 per 100,000 births; 95% CI 98–152 births) than in high-income countries (79 per 100,000 births; 95% CI 68–90 births) (*p* for interaction <0.01). While likely representing an underestimate, this model predicts that each year, nearly 400,000 new cases of pediatric hydrocephalus will develop worldwide [1]. In sub-Saharan Africa, its incidence is estimated at >180,000 infants yearly [2]. Treatment options available consist of either ventricular cerebrospinal fluid (CSF) shunts or endoscopic third ventriculostomy (ETV), which obviate the need for implants. Moreover, choroid plexus cauterization performed during ETV decreases CSF production and increases the procedure success rate for infants [3]. Mali is a country of 22 million inhabitants, an area of 1,241,000 km^2^ covered by 17 neurosurgeons working in 4 different centers across the country. However, due to the lack of equipment, endoscopic third ventriculostomy with cauterization of the plexus choroid (ETV/CPC) is only available in the capital, Bamako, which receives hydrocephalus infants from remote areas up to 1,500 km away. ETV/CPC offers advantages over traditional shunt treatment due to its affordability, the lower incidence of postoperative infections, and the overall lower long-term failure rate. Since the implementation of ETV/CPC in Mali (2013), no outcome data have been published. This paper aimed to report the author's experience with ETV/CPC since its introduction in Mali and enrich the body of literature regarding the outcome of ETV/CPC in sub-Saharan Africa.

## Materials and Method

### Study Design

**Fig. 1:**
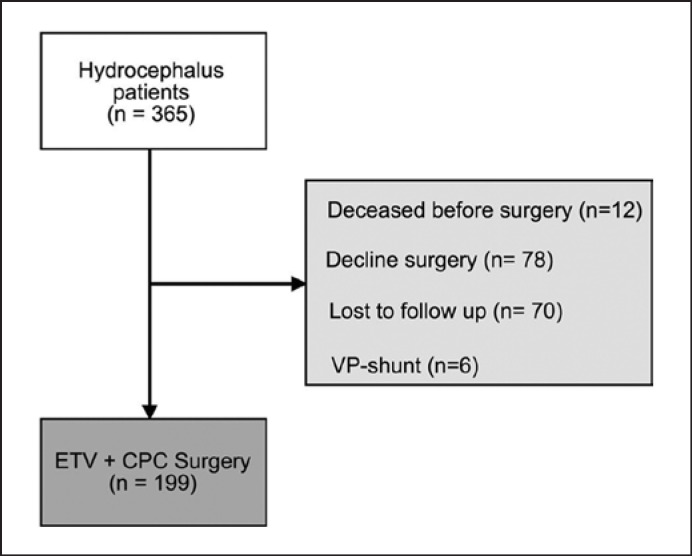
Diagram showing the selection criteria for the study population.

A retrospective study analyzed the outcome of hydrocephalus children aged under 12 months old who underwent ETV/CPC between July 2013 and January 2015 in Bamako, Mali, according to the CURE protocol as recently published by Lepard and coauthors (Table 1) [4]. One important difference from their protocol was that a shunt was not automatically placed when a scarred prepontine cistern was encountered at the time of the ETV, but rather, the procedure was given a chance to work. This series included 199 individuals out of 365 hydrocephalus children initially diagnosed and enrolled (Fig. 1). Enrolled patients who presented signs of infection such as fever, cough, or altered facies were transferred to the pediatric unit for further investigation and care. These patients were declared eligible for surgery once cleared by the pediatricians and had apyrexia for at least 7 days.

### Surgical Technique

**Fig. 2:**
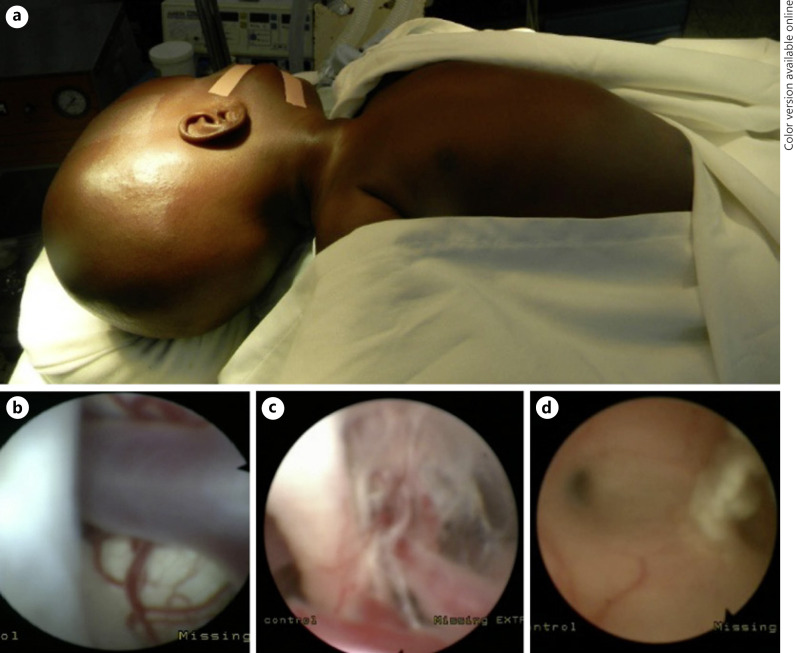
Per operative findings. **a** Positioning of the patient. **b** Visualization of the basilar trunk after opening the floor of the third ventricle. **c** Scarring tissue observed within the basal cisterns. **d** The aqueduct is obstructed (white arrows) by membrane deposits.

The surgery was performed as previously described [5]. Briefly, under general anesthesia and prophylactic antibiotic therapy with ceftriaxone before the incision, the patients were placed in a supine position with the head rotated left, placing the zygoma at the zenith. The thorax and abdomen were exposed in case of converting the procedure to a ventriculo-peritoneal shunt insertion (Fig. 2a). A paramedian 2-cm incision was made along the right corner of the anterior fontanelle. A 3.7-mm flexible endoscope was introduced into the right lateral ventricle followed by an exploration of the cavity. Classic anatomical landmarks such as the choroid plexus, thalamostriate veins, foramen of Monro, mammillary bodies, and the floor of the third ventricle were identified and the CSF was then taken for analysis. Next, the surgeon opened the floor of the third ventricle with the Bugbee wire without cautery when the floor is not hard, and sometimes with cautery when very hard, and then widened by stretching the Liliequist membrane. CSF flow through the opening was assessed. Subsequently, CPC was performed from the foramen of Monro around into the tip of the temporal horn on both sides, performing endoscopic septostomy as necessary. A final inspection of the ETV site was done before removing the endoscope. The skin was closed in a standard fashion after the closure of the dura mater. Postoperatively, the head circumference of patients was measured daily for 5 days before discharge.

All patients were followed in ambulatory care for at least 12 months, the time point when ETV Success score was graded. The mean follow-up was 16 months (range 13–21 months). Brain CT scans were ordered at 6 months and 12 months postsurgery. Patients with failed ETV/CPC underwent a second ETV procedure and if there was no apparent cause for failure, shunt placement was performed. A small number of patients underwent a second redo ETV.

### Study Approval and Consent to Participate

As a retrospective study using anonymized clinical data, this study design did not require informed written consent by the patients prior to conducting data collection. However, the study design was submitted and approved by the Local Institutional Review Board of “Hopital du Mali.”

### Statistical Analysis

Statistical analysis was conducted using Prism 9 GraphPad software. ETV successes were categorized according to the patient's age into 3 groups: ≤3 months, 3–6 months, and 6–12 months. Comparisons between groups were made using the ANOVA test followed by the Tukey test for comparison of means between each group. Statistical significance was defined at *p* < 0.05.

## Results

### Population Demographic

This series consisted of 119 males and 80 females. Patients were classified into 3 age groups: group 1 (age ≤3 months) included 80 patients (40%), group 2 (age between 3 months and 6 months) included 71 patients (36%), and group 3 (age between 6 months and 12 months) had 48 patients (24%). Fifty percent of patients had no prenatal care, including prenatal ultrasound. Also, 10% of patients were born at home. On physical examination, the head circumference ranged from 35 cm to 79 cm (mean = 42 cm), and 22 patients (11%) presented with decubitus ulcers on the scalp.

### Diagnosis and Etiology

There was fever, and sometimes seizure, at the time of presentation with hydrocephalus in 89 patients; however, only in 10% of them, CSF studies found bacteria: *E. coli* and *Staphylococcus aureus*. The remaining 110 patients were diagnosed at birth with macrocephaly, suggesting a congenital etiology. A brain CT scan was performed in all patients followed by Evans index assessment. The fourth ventricle and the lateral and third were enlarged for 131 patients (65%). Only the lateral and the third ventricles were enlarged in the remaining 68 patients (35%).

### Operative Management and Outcomes

The waiting time for surgery was on average between 2 and 3 weeks. Intraoperatively, the stigma of infection associated with anatomical distortion was noticed in 103 patients (52%). It consisted of hemosiderin deposit (8 patients), flaky deposit (11 patients), and distorted intraventricular anatomy in 82 patients (41%). Upon opening of the third ventricular floor, the basilar trunk was seen in all patients (Fig. 2b) with scar tissues around the basal cistern in 59 patients (32%) (Fig. 2c). In all these patients, ETV/CPC was carried out, followed by close monitoring. In case of failure (including failed repeat ETV), a shunt placement was performed. In those cases, with cisternal scarring, the failure rate was 80% (47 of 59 patients). During the surgery, there were 5 cases of significant bleeding. Exploration of the posterior part of the third ventricle found an aqueduct existing in 189 patients (95%). In 10 patients, the aqueduct appeared to be absent. Obstructive hydrocephalus was observed in 103 patients (52%) and a widened pineal recess (44%). In 34 patients, the aqueduct seemed patent on the CT scan but appeared closed by membranes at the time of surgery (Fig. 2d). The typical ETV/CPC operative time ranged from 90 to 120 min.

The overall success rate at 12-month follow-up (including those who required repeat ETV) was 47% (94 patients). The success rate was 69% in the 6 ≤ 12-month age group (33 patients), 54% in the 3 ≤ 6 months age group (38 patients), and 29% in those ≤3 months of age (23 patients). Repeat ETV was performed in the cases of failure from ETV closure (Table 2). Postoperative meningitis occurred in 2 cases that were treated adequately with antibiotics. Sixteen (16) patients (8%) died within 6 months of the operation, and the causes were severe malaria for 6 patients, severe pneumopathy for 5 patients, and nonspecific infectious syndrome for 5 patients.

### Comparison of ETV Outcomes between Groups

ETV outcomes differed according to the patient's age (one-way ANOVA, *F* = 4.31, *p* = 0.0099). In this study, patients were categorized in 3 groups: ≤3 months (80 patients), between 3 and 6 months (71 patients), and between 6 and 12 months (48 patients). The success rates were 29% in group 1, 54% in group 2, and 69% in group 3. Using Tukey test, following one-way ANOVA, we found a significant difference between group 1 and group 3 (*p* = 0.007).

## Discussion

Hydrocephalus is a major concern in developing countries. Given the geographical conditions (1,500 km between certain regions and the appropriate healthcare centers) and safety conditions (country at war) in Mali, the need for affordable procedures with limited surveillance is essential. Thanks to the support of CURE International, ETV/CPC became available to pediatric hydrocephalus patients in the country in 2013.

### Demographic Considerations

Studies from the African literature reported a male predominance of pediatric hydrocephalus in Tanzania, Uganda, and Nigeria [5], which was not corroborated in our study. This may be due to a sample bias in our series. The most common age group at presentation was 0–3 months (40%) followed by 4–6 months (36%). We did not find a correlation between age and perioperative complications as described in some studies [6, 7]. However, there was a correlation between age and the success rate of the procedure [8].

### Clinical Presentation

The head circumference is an indicator of the severity or delay in the diagnosis of hydrocephalus [9]. The delay in patient care observed here is explained not only by the difficulty to access the neurosurgical healthcare unit but also by the involvement of traditional healers who most often receive the patients first. This illustrates the misconception about hydrocephalus in many sub-Saharan countries. Parents assume hydrocephalus could be a curse or a disease curable by traditional healers. Hence, many children are referred to the hospitals in a state of dehydration, poor nutrition, sunset gaze, and bulging fontanelle.

### Etiologies of Hydrocephalus

Two main etiologies prevail in pediatric hydrocephalus in sub-Saharan Africa: infection (congenital or acquired) and malformation [10, 11]^.^ The etiologic investigation can be challenging in this low-income country where prenatal care is inconstant or incompletely rendered. As illustrated in our study, prenatal ultrasounds were performed in only 40% of cases. The neurological form of malaria is a prevalent comorbidity in Mali that may delay the diagnosis and treatment of bacterial meningitis. Most children were treated with antimalarial drugs and antibiotics at the same time before surgery. In some instances, intraoperative identification of hemosiderin deposits led us to consider ventricular hemorrhage as the etiology of hydrocephalus, although such deposits are also common following infectious ventriculitis.

### Preoperative Imaging

Imaging studies are essential for diagnosis and preoperative strategy. All our patients underwent a brain CT scan that allowed us to assess the extent of ventricular dilatation and patency of the mesencephalic aqueduct. Tetraventricular hydrocephalus was noted in 65% and triventricular in 35% in our series, but, during surgery, endoscopic findings noted an obstructed aqueduct in 52%. In the Ugandan series, two-thirds of children with postinfectious hydrocephalus were noted to have an obstructed aqueduct at the time of ETV [12].

### CSF Study and ETV/CPC Procedure

CSF was systematically sampled from a transfontanelle approach to rule out or treat meningitis. Yet, in this low-resource environment, microbiological analysis was not always affordable [2]. Hence, in case of high suspicion of meningitis, patients were treated empirically with ceftriaxone 1 g/day for 10–14 days.

Routinely, the surgeons check the aqueduct patency during ETV/CPC procedures. In 34 cases, the aqueduct was obstructed by pseudomembranes in patients previously diagnosed by CT scan as having tetraventricular (communicating) hydrocephalus. This transformation from communicating to obstructive hydrocephalus could be explained by the delay between diagnosis and surgery associated with subsequent scarring posthemorrhage or postinfection that obstructs the aqueduct [13]. Findings of hemosiderin deposits during the inspection of ventricular cavities allowed us to subsequently reconsider the etiology of hydrocephalus mistakenly accounted as congenital. Previous studies have reported that re-canalization of the aqueduct increased ETV success score [14]. Yet, the presence of scarring tissues in the basal cistern is associated with a decreased success of the procedure [15].

### Complications

Complications of this procedure are dominated by perioperative intraventricular hemorrhage [14]. During surgery, 5 patients in our series had bleeding requiring active irrigation. Rigorous aseptic preparation is mandatory to avoid infection. Protocols for reducing post-shunt meningitis are also implemented in ETV [15]. In a recent study on sub-Saharan African children, Kalangu et al. [16] reported a 1.9% incidence of post-shunt meningitis. Besides limiting the traffic and individuals present in the operating theater, continuous skin application of 10% povidone-iodine solution throughout the procedure was key to achieving this low incidence. The infection rate was 1% in our series. We observed an 8% mortality that was not directly related to the procedure but was still higher than the literature data [17].

### Management of Failed ETV/CPC Procedure

In the case of recurrent hydrocephalus after ETV/CPC procedure, a re-intervention was performed to investigate the causes of failure. ETV/CPC could fail in case of secondary closure of the third ventricle floor by membrane formation in our series. In some instances, the third ventricle floor may be open but the Liliequist membrane became closed afterward. These observations were found in the series by Marano et al. [18].

Repeating ETV can be an effective treatment for over 50% of failed ETV/CPC. In the largest published experience, those with the longest time to failure of initial ETV/CPC >6 months had a 91% success rate, 3–6 months 60%, and <3 months 42% [18]. As corroborated by the literature, we experienced that repeated ETVs were more successful with patients over 3 months old [19]. Early failure ETV/CPC (less than a week) is a pejorative factor for a repeated procedure. In one study, reopening the floor of the third ventricle was successful in 50% of early failures versus 78% of later failures (after 7 days of initial ETV) [20]. In our series, 94 patients were able to avoid shunt placement, including those requiring repeat ETV, as described in Table 2. Systematic coagulation of the choroid plexus was performed to increase the success of ETV as reported in the literature [5]. Our success rate of 47% was adversely affected by the predominance of the youngest age group (1st group, less than 3 months) and by our not shunting those found to have significant cisternal scarring at the time of the ETV, as in the prospective studies from Nigeria and Uganda reported by Lepard et al. [4]. Nonetheless, similar to their results, we were able to successfully avoid shunt dependence in nearly half of all infants presenting with hydrocephalus at less than 1 year of age by employing ETV/CPC as the preferred treatment, including all etiologies, those with a cisternal scar, and those under the age of 3 months.

## Conclusion

The endoscopy is an invaluable tool for the management of hydrocephalus. Our findings suggest that although age is an important factor in predicting ETV success, ETV/CPC is a safe and effective technique in Mali to manage hydrocephalus in children under 12 months of age. In low-income countries, this procedure reduces the use of implanted shunts and helps alleviate the danger of shunt dependence for those with limited access to emergency neurosurgical management.

## Statement of Ethics

Ethical approval and consent were not required as this study was based on publicly available data.

## Conflict of Interest Statement

The senior author Oumar Diallo was a fellow of Cure International and has received endoscopic equipment from this non-profit organization. The co-authors have no conflict of interest to declare.

## Funding Sources

The authors of the present study did not receive any financial support or sponsorship.

## Author Contributions

Oumar Diallo: conceptualization, methodology, data curation, formal analysis, writing, and review. Mahamadou Dama: data Acquisition and image editing. Landry Konan: writing draft, editing, and proofreading. Oumar Coulibaly: data acquisition and data curation. Daouda Sissoko: data acquisition and writing original draft. Abdoulaye Hima Maiga: review and approval of final version before submission.

## Data Availability Statement

Raw data generated or analyzed during this study are uploaded and available at https://osf.io/haeyt/. Nonetheless, further inquiries can be directed to the corresponding author.

## Figures and Tables

**Table 1:** Protocol at CURE Children's Hospital of Uganda (CCHU), Mbale, Uganda

	Type A <1 year old AQ, open	Type B ≥1 year old AQ, open	Type C <1 year old AQ, closed	Type D ≥1 year old AQ, closed
PIH	ETV/CPC	ETV/CPC	ETV	ETV/CPC
NPIH	ETV/CPC	ETV/CPC	ETV/CPC	ETV
MM	ETV/CPC			

AQ, aqueduct; CPC, choroid plexus cauterization; ETV, endoscopic third ventriculostomy; MM, myelomeningocele; PIH, postinfectious hydrocephalus; NPIH, non-postinfectious hydrocephalus.

**Table 2:** Breakdown of patients by procedure and age

Age/Success	First ETV/CPC, %	Second ETV, %	Third ETV, %
1–3 months	26	28	29
4–6 months	44	49	54
7–12 months	54	63	69

AQ, aqueduct; CPC, choroid plexus cauterization; ETV, endoscopic third ventriculostomy.
